# Extensive loss of forage diversity in social bees owing to flower constancy in simulated environments

**DOI:** 10.1098/rspb.2024.1036

**Published:** 2024-07-31

**Authors:** Christoph Grüter, Francisca H. I. D. Segers, Lucy Hayes

**Affiliations:** ^1^ School of Biological Sciences, University of Bristol, 24 Tyndall Avenue, Bristol BS8 1TQ, UK

**Keywords:** flower constancy, agent-based models, communication, nutrition, diet, bees

## Abstract

Many bees visit just one flower species during a foraging trip, i.e. they show flower constancy. Flower constancy is important for plant reproduction but it could lead to an unbalanced diet, especially in biodiversity-depleted landscapes. It is assumed that flower constancy does not reduce dietary diversity in social bees, such as honeybees or bumblebees, but this has not yet been tested. We used computer simulations to investigate the effects of flower constancy on colony diet in plant species-rich and species-poor landscapes. We also explored if communication about food sources, which is used by many social bees, further reduces forage diversity. Our simulations reveal an extensive loss of forage diversity owing to flower constancy in both species-rich and species-poor environments. Small flower-constant colonies often discovered only 30–50% of all available plant species, thereby increasing the risk of nutritional deficiencies. Communication often interacted with flower constancy to reduce forage diversity further. Finally, we found that food source clustering, but not habitat fragmentation impaired dietary diversity. These findings highlight the nutritional challenges flower-constant bees face in different landscapes and they can aid in the design of measures to increase forage diversity and improve bee nutrition in human-modified landscapes.

## 1. Introduction

Bees are essential pollinators of wild and agricultural plants [1–3], owing to their abundance and their morphological and behavioural diversity [4,5]. Another key reason for their value as pollinators is their diet. Many bees have a broad (i.e. polylectic) diet at the species level, but as individuals they specialize on one flower species during a foraging trip, the so-called flower constancy [6–10]. Flower constancy is beneficial for plant fitness as it reduces conspecific pollen loss and the negative impacts of heterospecific pollen deposition [7,10–13]. However, the causes and consequences of flower constancy from the bees’ perspective remain less well understood [7,9,14]. One benefit of flower constancy may be that it avoids the time and cognitive load associated with having to learn how to exploit multiple flower species efficiently [7–10,14–16]. However, it remains a puzzling behaviour as both individual bees and different bee species vary in the degree of flower constancy [9,10,17,18].

Bees require a balanced diet to maintain basic biological functions [19–23]. Pollen, in particular, plays a key role in bee health as the main source of protein, lipids and micronutrients [19,20,22]. An inadequate supply of protein (and essential amino acids) has been shown to impair body size [24–26], lifespan [27,28] and ovary development [27] in both social and solitary bees. Pollen from different plant species vary greatly in their macro- and micro-nutrient content [21,22,29,30], and a diet based on a small number of pollen types risks a surplus of some nutrients and a deficiency in others [31], with negative impacts on the reproductive success [32] and survival [28,33,34] in *Bombus terrestris* and *Apis mellifera*. Accordingly, there is increasing evidence from different bee species that a more diverse pollen diet has health benefits [35], for example, by boosting larval size [36], colony growth [37,38], immunocompetence [39] and the survival of bees infected by viruses or parasites [30,33,40]. Flower constancy, which can last several days [41,42], could negatively impact dietary diversity and exacerbate the effects of biodiversity loss in strongly human-modified environments, such as agricultural landscapes. Foraging challenges in modern landscapes are suspected to be a key driver of poor bee health as environments lacking in floral diversity make it harder for bees to achieve their intake targets for important nutrients [22,23,30,31,38,43–45]. Evidence for the combined effects of environmental change and narrow dietary preferences comes from observations showing the bumblebees with a narrow diet were more likely to decline in numbers in the last decades [46]. Yet, if and how flower constancy affects dietary diversity under different ecological circumstances remains unknown.

Social bees, mainly the honeybees (Apini), bumblebees (Bombini) and stingless bees (Meliponini), are often highly flower constant [17,47]. It is assumed that flower constancy does not negatively affect forage diversity in social bees because different individuals can specialize in visiting different flower species, thereby ensuring that the colony exploits a range of plant species [14,48,49]. While social bee colonies indeed collect pollen from many plant species, often only approximately 1–5 pollen types are collected in larger amounts at any given time [19,50–53], possibly risking nutritional deficiencies. Colony size could be a key factor mediating the effects of flower constancy on colony forage diversity as a larger foraging workforce could potentially discover a wider diversity of plant species than a smaller one. Another social trait with potential implications for forage diversity is recruitment communication. Many social bees communicate about profitable food sources [54–56], e.g. the honeybee waggle dance, excitatory runs in combination with buzzing sounds in bumblebees and stingless bees or trophallaxis in honeybees and stingless bees [54,55,57,58]. The function of these diverse behaviours is to direct nestmates towards profitable food sources, often by transmitting olfactory information that allows recruits to identify the advertised plant species in the surrounding environment [54,59,60]. Recruitment communication may reduce colony forage diversity because it causes colonies to focus on a subset of the available food sources [57,61,62].

Our understanding of the consequences of flower constancy and communication on colony diet breadth remains limited, first, because it is not usually possible to manipulate flower constancy while keeping other factors constant and, second, because it is logistically challenging to perform flower constancy experiments at a landscape scale. Agent-based simulation models are powerful tools to circumvent these obstacles and provide insights into how social and ecological factors interact with foraging strategies to modify emergent colony-level properties [14,63–67]. We developed an agent-based simulation model to study if and how flower constancy and communication affect the diversity of plant species collected by a colony in both plant species-rich and species-poor environments. Within these environments, we manipulated food source distribution (uniform versus clustered), abundance and reward size. In addition, we created fragmented landscapes, e.g. representing an urban habitat, to explore how habitat fragmentation affects forage diversity with a view to help in the design of conservation strategies which could improve bee nutritional diversity in human-modified landscapes. We measured both *species diversity*, i.e. the number of plant species a colony discovers, and *Simpson’s diversity*, which also takes into account the evenness of plant species exploitation [68]. We predicted that colony size, plant species richness and food source distribution (clustered versus uniform) determine the effects of flower constancy and communication on dietary diversity. Finally, we performed a literature search to assess the degree of flower constancy in bees with different social lifestyles to aid in the interpretation of our simulation results.

## 2. Material and methods

### (a) Agent-based model

We built an agent-based model (ABM) using the programming software NetLogo 6.1 [69] (see NetLogo files with full model code [70]). The model builds on an earlier version [14,63], from which it differs in key aspects, such as the types of data that were collected, the way food sources were distributed, their characteristics and the number of flower species in the environment. The model simulates a bee colony surrounded by food sources (see §2c for descriptions of food sources and electronic supplementary material, table S1 for default and alternative values tested). The model does not simulate a particular bee species but is built to resemble a bumblebee or a *Melipona* stingless bee colony in terms of colony size, flight behaviour and communication mode. The bees operate on a two-dimensional square grid with 400 × 400 patches. A single patch length corresponds to 5 m. Thus, the size of the virtual world corresponds to 2 ×2 km, which covers the typical foraging distances of most bee species [63,71,72] (electronic supplementary material, figure S1). Each simulation lasted 36 000 s (i.e. 10 h in total), representing a day with good foraging conditions.

### (b) Simulated forager bees

Colony sizes ranged from 10 to 300 bees, which covers the typical forager workforces of many bumblebee and stingless bee species [73,74]. Bees began the simulation in the centre of the nest as *generalists* (electronic supplementary material, figure S2). They then moved at a flying speed of 1 patch s^−1^ (*v*_flight_), a flight speed similar to that of bumblebees (5 m s^−1^) [75], following a Lévy-flight pattern [76,77]. A Lévy flight is a random sequence of flight segments whose lengths, *l*, come from a probability distribution function having a power-law tail, *P*(*l*) ~ *l* ^−μ^, with 1 < *μ* < 3 (with *μ* = 1.8 as our default. This is within the range found in bumblebees, see [77] for a discussion of Lévy-flight patterns in different bees and contexts). After agents encountered a food source, they remained on the patch for an average duration of 600 ± 120 s (*t*_flower-stay_, mean ± s.d.), simulating a bee visiting a small group of flowers of the same species rather than an individual flower. Agents searched for food sources until they were full, after which they returned to the nest for unloading. They stayed in the nest for 300 s (*t*_nest-stay_) [41]. The speed of agents moving inside the nest (*v*_nest_) was 0.1 (patch s^−1^), which allowed them to encounter and recruit other agents (see §2e).

### (c) Food sources

Either 12 (species-rich environment) or four (species-poor environment) different flower species were in the environment. Foragers needed to visit either two (large rewards) or 10 (small rewards) food sources to fill up. In species-rich environments, the number of food sources (*FS*_number_) per flower species was a random number between 0 and 200 (low abundance; *FS*_numberLow_, mean = 100 per species) or between 0 and 2000 (high abundance; *FS*_numberHigh_, mean = 1000 per species). In species-poor environments, it was a random number between 0 and 600 (low abundance; *FS*_numberLow_, mean = 300 per species) or between 0 and 6000 (high abundance; *FS*_numberHigh_, mean = 3000 per species). Thus, flower species-rich and species-poor environments differed in the number of flower species, but not in the number of food sources. The distribution of food sources in the environment was either uniformly random or clustered (electronic supplementary material, figure S1*a*,*b*). When food sources were clustered, we simulated 10 clusters per flower species (default) at a moderate clustering strength (see electronic supplementary material, figure S1*b*). We also tested environments with 30 clusters, i.e. clusters were three times more numerous but smaller.

To test if habitat fragmentation affects forage diversity, we simulated a fragmented environment that contained four or eight ‘build-up’ areas, comprising 36% and 50% of the total foraging area, where no food sources were available (thus, 36% or 50% fewer food sources, respectively). This could represent areas with buildings, empty crop fields or roads (electronic supplementary material, figure S1*c*,*d*).

We tested different refill times (*t*_refill_) for food sources after visits: 0, 1200 (default) and 3600 s [78]. When *t*_refill_ = 0, food sources became rewarding again immediately after the visit of a bee. With *t*_refill_ = 1200, a food source remained unrewarding for the equivalent of 20 min after it had been visited by a bee, leading to exploitation competition between nestmates.

### (d) Flower constancy

The degree of flower constancy of bees during foraging trips depends on a range of extrinsic and intrinsic factors in nature [7,9] but is close to 100% in some eusocial bee species [42,50,79]. In our model, bees visited food sources either indiscriminately (random choice) or they were strictly flower constant, i.e. they remained faithful to the flower species they discovered first on their initial foraging trip.

### (e) Recruitment communication

Social bees use different behavioural mechanisms to transmit information about high-quality food sources, such as their odour or location, and thereby bias the food source preferences of their nestmates towards more profitable options [54–57,80]. The model simulated a generic process that allows foragers that have visited a high-quality flower species to bias the food preferences of nestmates during encounters inside the nest (*influencers*). To this end, three out of 12 (species-rich environment) or one out of four (species-poor environment) flower species were designated to be of higher quality, and foragers visiting these high-quality flower species could become *influencers* upon return to the nest. *Influencers* recruited other agents that were not flower constant to the high-quality flower species by changing the latter’s preference if they encountered them on the same patch inside the nest. Following such an encounter, recruited agents would leave the nest to search for food sources of the corresponding high-quality flower species. Since the motivation to show communication behaviours often decreases with increasing food source distance [57,73], the probability of becoming an *influencer* decreased with increasing distance of the last visited high-quality food source (electronic supplementary material, figure S3*a*).

#### (i) Measured variables

To assess how the different parameters affect the dietary diversity of a colony, we measured the total number of flower species visited by the foragers of a colony during a simulation (*species diversity*). Since forage diversity also depends on the relative abundance (or evenness) of visits, we also calculated the *Simpson’s diversity index* (*SDI*) to assess how evenly the different flower species were collected by a colony. The *SDI* was measured as 1 – *D*, where: $D=\sum n_{i}\left(n_{i}-1\right)/N\left(N-1\right)$ , with *n_i_* = the number of food sources in the *i*th flower species and *n* = the total number of food sources. The *SDI* varies between 0 and 1, with a higher score indicating a higher diversity and evenness of visited flower species.

We tested if *species diversity* and the *SDI* depended on flower constancy (versus indiscriminate choice), colony size, communication (versus no communication), the total abundance of food sources, and their distribution in flower species-rich and species-poor environments. We performed 10 runs for each parameter combination. We did not calculate statistical *p*-values owing to the arbitrariness of the simulation number but indicated 95% confidence intervals for effect sizes.

### (f) Sensitivity analysis and model exploration

In addition to the factors mentioned above, we explored several other factors and how they affected our results. These included Lévy flight *μ*, refill time, relative reward sizes (larger for high-quality flower species) or the number of high-quality flower species (electronic supplementary material, table S1).

### (g) Pollen load purity data from the literature

We searched the published literature for information on the strength of flower constancy in different bee species (relying mainly on [17,35,47,50,79]. We included a species if at least 20 bees were sampled and the proportion of bees that collected pure pollen loads (>97% pollen grains belonging to one species) was reported (electronic supplementary material, table S2). We analysed the effects of lifestyle (as classified by Michener [4]: ‘highly eusocial’ = perennial lifestyle with extensive morphological differences between queen and workers; ‘primitively eusocial’ = annual lifestyle and only a moderate morphological difference between queen and workers and solitary) on flower constancy using phylogenetic generalized least-squares (PGLS) models [81]. The phylogenetic framework for the PGLS models relied on phylogenetic trees with branch lengths corresponding to geological time, based on trees for Anthophila [82], Andrenidae [83], Meliponini [84], *Melipona* [85], *Bombus* [86] and *Osmia* [87]. A tree was created by pruning species to include only the taxa relevant for the comparative analysis (see electronic supplementary material, figure S3*b*).

## 3. Results

### (a) Flower species-rich environment—small rewards

**Figure 1.**
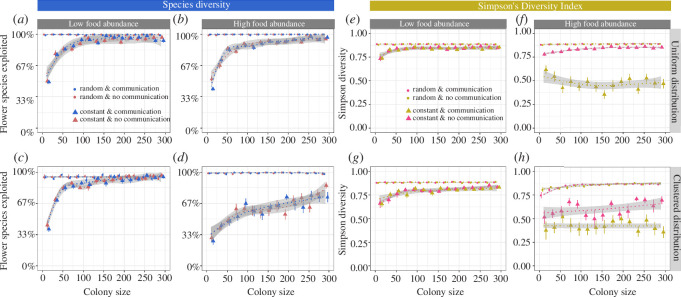
The *species diversity* (as the percentage of all available flower species) (*a–d*) and *Simpson’s diversity index (SDI*) (*e–h*) in relation to colony size, food source distribution, food abundance and foraging strategy (flower constancy and communication) when food sources offered *small* rewards and needed time to replenish (1200 s). Colonies were either flower constant (triangle) or foraged indiscriminately (= randomly, circles); colonies either had communication (blue in (*a–d*); pink in (*e–h*)) or consisted of bees that foraged solitarily (red in (*a–d*); orange in (*e–h*)). *Uniform distribution* means that food sources were uniformly distributed, whereas *clustered distribution* means that food sources were clustered. Ten clusters per plant species were simulated (default, see §2 for more details).

Flower constancy led to a loss of colony forage diversity (figure 1*a–d*), especially in small colonies: when flower-constant colonies consisted of 10 foragers, colonies exploited only approximately 30–50% of the available plant species (figure 1*a–d*), compared to approximately 100% exploited by indiscriminate colonies. As predicted, the percentage of flower species exploited by flower-constant bees increased with colony size. When food sources were abundant and clustered (figure 1*d*), however, even large, flower-constant colonies (>250 bees) exploited only 60–70% of the available plant species.

The *SDI* was also lower in flower-constant colonies compared to indiscriminate colonies (figure 1*e–h*). However, the *SDI* was not greatly affected by colony size. On the other hand, there was a pronounced negative effect of communication on the *SDI* in environments with high food source abundance, even in large colonies (figure 1*e,g* versus 1*f,h*). For example, when food sources were clustered and colony sizes large (>250 bees), approximately 75% of all foraging trips were to just one flower species (electronic supplementary material), predominantly a high-quality one.

The reported outcomes refer to food sources that needed time to replenish after a visit (refill = 1200 s). We also explored forage diversity when food sources replenished immediately after a visit (refill = 0 s), thereby removing exploitation competition. The overall patterns were very similar (electronic supplementary material, figure S4).

### (b) Flower species-rich environment—large rewards

**Figure 2. F2:**
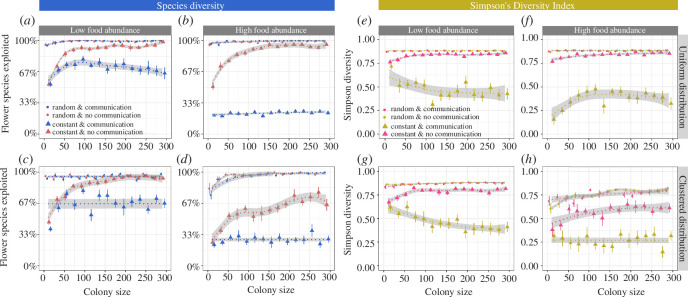
The *species diversity* (as the percentage of all available flower species) (*a–d*) and *SDI* (*e–h*) in relation to colony size, food source distribution, food abundance and foraging strategy (flower constancy and communication) when food sources offered *large* rewards and needed time to replenish (1200 s). For further explanation, see the legend of figure 1.

When food sources offered large rewards, the negative effects of flower constancy on *species diversity* were even more pronounced, especially when colonies could also communicate about high-quality flower species (figure 2*a–d*), as increasing colony sizes no longer mitigated the negative impact of flower constancy. For instance, when food source abundance was high (figure 2*b,d*), flower-constant colonies with communication discovered and exploited only approximately 20–30% of the available plant species, irrespective of colony size. The overall patterns were similar when food sources replenished immediately after a visit (electronic supplementary material, figure S5).

The *SDI* was again lower in flower-constant colonies (figure 2*e–h*) and communication caused flower-constant colonies to focus most of their attention on one or two high-quality plant species. For instance, when food sources were abundant and clustered, approximately 84% of all foraging trips of large (>250 bees), flower-constant colonies with communication were to just one high-quality plant species (electronic supplementary material).

### (c) Flower species-poor environment—small rewards

**Figure 3.**
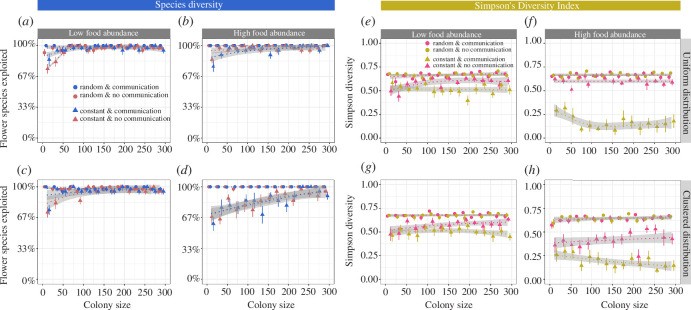
The *species diversity* (as the percentage of all available flower species) (*a–d*) and *SDI* (*e–h*) in relation to colony size, food source distribution, food abundance and foraging strategy (flower constancy and communication) in environments with low plant diversity (four species), when food sources offered *small* rewards and needed time to replenish (1200 s). For further explanation, see the legend of figure 1.

We also explored the effects of flower constancy and communication in environments with low plant diversity (four plant species). The effects of flower constancy were again similar to what we found in more diverse environments (figure 3*a–d*). However, the effect of colony size was less strong in relative terms: small, flower-constant colonies exploited approximately 70–80% of all available plant species ( approx. three flower species), compared to approximately 30–50% in a flower species-rich environment (approx. 4–6 flower species). The loss of forage diversity in flower-constant colonies was again more pronounced when food sources were abundant and clustered (figure 3*c,d*).

The *SDI*, on the other hand, showed a contrasting pattern and was generally lower in environments with a low plant diversity (figure 3*e–h*) compared to a flower species-rich environment, revealing a strongly uneven exploitation of food sources. For example, when food sources were clustered, large (>250 foragers) flower-constant colonies with communication performed approximately 91% of all foraging trips to just one plant species (electronic supplementary material). The overall patterns were similar when food sources replenished immediately after a visit (electronic supplementary material, figure S6).

### (d) Flower species-poor environment—large rewards

**Figure 4.**
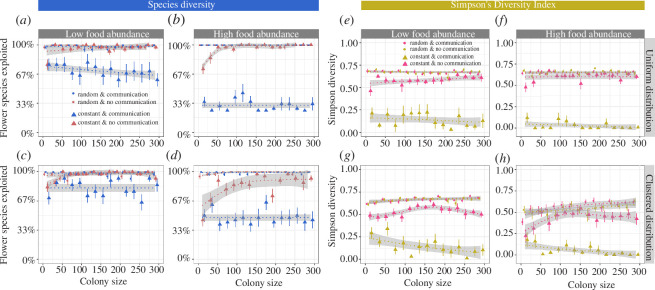
The *species diversity* (as the percentage of all available flower species) (*a–d*) and *SDI* (*e–h*) in relation to colony size, food source distribution, food abundance and foraging strategy (flower constancy and communication). All measurements are from environments with low plant diversity (four species), in which food sources offered *large* rewards and needed time to replenish (1200 s). For further explanation, see the legend of figure 1.

*Species diversity* was again much more impacted by flower constancy and communication when food rewards were large (figure 4*a–d*) compared to when rewards were small (figure 3*a–d*). This effect was especially strong when food source abundance was high (figure 4*b,d*). Under these conditions, flower-constant colonies that used communication incorporated only 1–2 of the four available flower species into their diet (figure 4*b,d*).

The *SDI* approached zero in high food abundance environments and when flower-constant colonies also used communication (figure 4*f,h*), meaning that just one flower species was exploited. The overall patterns remained similar when food sources replenished immediately after a visit (electronic supplementary material, figure S7).

### (e) Fragmented landscapes and different types of clustering

We simulated colonies in two different fragmented landscapes, mimicking urban and agricultural habitats (electronic supplementary material, figure S1*c*,*d*). Fragmented environments offered fewer food sources since they were partly covered by squared (covering 36% of the total area) or striped (50% of the total area) areas without food. As a result, colonies collected less food in these environments (electronic supplementary material; approx. 18 and 23% fewer food source visits in environments fragmented by squares or stripes, respectively).

**Figure 5.**
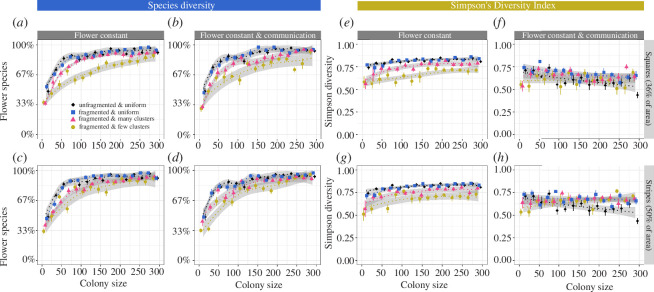
The *species diversity* (as the percentage of all available flower species) (*a–d*) and *SDI* (*e–h*) in relation to colony size, type of fragmentation (squares versus stripes versus unfragmented; see electronic supplementary material, figure S1*c,d*) and foraging strategy (flower constancy and communication). All measurements are from environments with high flower species diversity (12 species), in which uniformly distributed food sources offered *small* rewards and needed time to replenish (1200 s). Food source abundance was intermediate in all cases, with an average of 500 food sources per plant species (approx. 6000 in total).

We tested if two types of clustering, few large clusters versus many small clusters, differentially affected forage diversity. Unexpectedly, *species diversity* and the *SDI* were similar in fragmented versus unfragmented environments (figure 5). A noteworthy exception is that, when colonies could communicate, large colonies collected food with a lower *SDI* in unfragmented landscapes than in unfragmented landscapes (figure 5 *f,h*). Possibly, communication about high-quality flower species was faster in unfragmented environments with more abundant food sources as bees encountered food sources quicker, causing a narrower diet. Forage diversity was overall higher when clusters were smaller but more numerous (30 clusters) than when clusters were larger but fewer in number (10 clusters) (figure 5).

### (f) Sensitivity analysis and model exploration

In addition to the parameters discussed above, we tested the effects of several other factors and parameters, including food source number (maximum of 50, 100, 500 and 1000 per plant species) and longer refill times (3600 s). We also tested environments where only one flower species was of high quality and situations when high-quality flower species offered more voluminous rewards than low-quality flower species (50% versus 10% of the load capacity) and we simulated alternative Lévy-flight *μ* values (to 1.4 and 2.4). The general patterns were similar (data can be found in the electronic supplementary material). A previous version of the model found that changing recruitment parameters (electronic supplementary material, figure S3*a*) had no noticeable effect on the visitation rates of different flower species [14] and, therefore, these were not explored.

### (g) Pollen load purity

**Figure 6.**
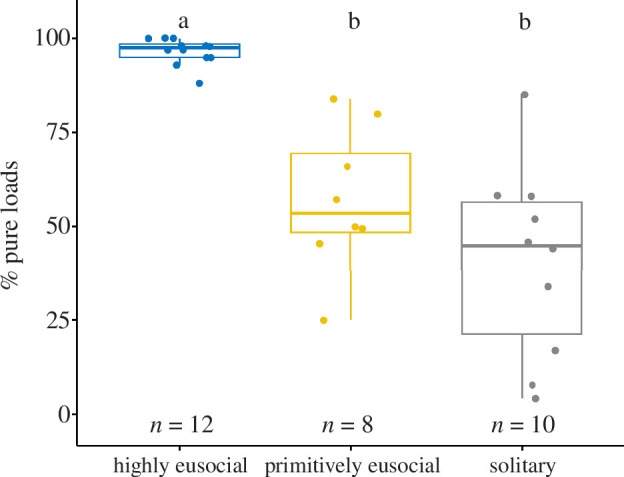
Percentage of foragers collecting pure pollen loads in 30 bee species. Letters a and b indicate statistically significant differences (see electronic supplementary material, table S2 for details).

Our comparative analysis included 30 bee species belonging to five bee families (electronic supplementary material, table S2). Foragers of highly eusocial species (Apini and Meliponini) were more likely to collect pure pollen loads (96.6 ± 3.5% pure loads, *n* = 12 species) than primitively eusocial species (*Bombus* and a *Lasioglossum*; 57.1 ± 19.3%, *n* = 8 species; PGLS: *t* = −2.3, *p* = 0.029) and solitary species (40.6 ± 25.3%, *n* = 10 species*; t* = −2.59, *p* = 0.015), suggesting that highly eusocial bee species have the highest degree of flower constancy. There was no significant difference between primitively eusocial and solitary species in how often bees collected pure pollen loads (*t* = −0.8, *p* = 0.43) (figure 6; electronic supplementary material, table S2).

## 4. Discussion

Flower-constant colonies discovered and exploited only a subset of the available flower species under most conditions, unlike colonies with indiscriminate foragers which exploited close to 100% of all available flower species. Especially small, flower-constant colonies often exploited only 30–50% of the available flower species. The *SDI*, a measure of how evenly colonies exploited different flower species, was particularly low if flower-constant colonies also used communication about high-quality flower species, even when colony sizes were large. Under these conditions, colonies visited predominantly just one or two flower species of higher quality. This is consistent with observations in *Scaptotrigona* stingless bees, which have large colony sizes and efficient recruitment communication [88,89]: Ramalho [53] observed that while colonies collected pollen from a range of plant species, they mainly concentrated on *Eucalyptus* pollen. Similarly, honeybee (*Apis mellifera*) colonies have been observed to concentrate on highly profitable plant species even if patches are found at distances of several kilometres [90]. The ability to focus on profitable flower species is a crucial benefit of communication [54,57,61], but it could jeopardize a diverse diet. Collecting only a small number of pollen types increases the risk of missing nutritional intake targets, e.g. by collecting pollen with a low protein content [24,28,32,36,91] or collecting large quantities of pollen containing harmful compounds, such as toxic phytochemicals [35]. On the other hand, the high *species diversity* found in larger flower-constant colonies suggests that colony size can help colonies avoid deficiencies in micronutrients, i.e. nutrients that are required only in small quantities, even if *Simpson’s diversity* is low. Whether plant *species diversity* or *SDI* is a more relevant measure of forage diversity is likely to depend on the nutrient type and whether bees require large or small amounts of particular nutrients, which remains unknown for most bee species [23,31].

Colonies with only 10 foragers discovered only a fraction of the available flower species. Temperate bumblebee colonies have a handful of pollen foragers in spring and even a mature colony of buff-tailed bumblebees (*Bombus terrestris*) or common carder bees (*Bombus pascuorum*) with 100–150 workers [74,92] contains a relatively small number of pollen foragers (approx. 15–20% of the colony) [41,93]. Many highly eusocial bees, like honeybees or stingless bees, will have several hundred to a few thousand active foragers [56,73] and are, therefore, predicted to discover a larger number of flower species (*species diversity*). A higher risk of missing out on key nutrients might explain why bumblebees and solitary bees are less flower constant than honeybees and stingless bees (figure 6) [47,48] and, in turn, why bumblebee colonies often collect pollen from a larger number of flower species than honey bee colonies in the same area [50,94]. Differences in the risk of an unbalanced nutrient intake could also explain why bumblebees, but not honeybees, can discriminate between pollen types based on protein and lipid content [95–98]. Honeybees and stingless bees, on the other hand, store pollen inside the nest for longer time periods, which could help bees access a larger diversity of pollen types kept in storage [99] and could allow nurse bees to actively mix pollen types during the preparation of brood food. Whether and how nurse bees responsible for preparing brood food mix stored pollen to create a balanced diet is an intriguing unanswered question.

Simulations suggest that the effects of flower constancy and communication on forage diversity also depend on ecological factors, such as the distribution and abundance of food sources. Paradoxically, forage diversity was often lower when food sources were more abundant (see e.g. figure 1). Increased food source abundance means that food sources are discovered quicker, potentially ‘locking’ colonies with flower constancy and communication into collecting certain types of food more rapidly. This effect was amplified by food source clustering, which could mimic large patches or fields of a flower species (see figures 1d–3d [Fig F2]–3). Food source clusters in nest proximity are likely to be discovered first, which will bias the foraging preferences of a flower-constant colony towards this flower species.

When rewards were large, colonies with both flower constancy and communication experienced extremely low forage diversity, both in *species diversity* and in the *SDI*. One explanation is that foragers collecting larger rewards needed to visit fewer food sources to fill up (e.g. the pollen basket), thereby reducing the amount of time spent searching for food sources and increasing the time spent inside their nest. This is likely to amplify the effects of communication by increasing opportunities for *influencers* to bias the foraging preferences of nestmates towards high-quality flower species, an effect that could be amplified further by the tendency of bees to be more flower constant when the rewards they experience are larger [100].

Humans have drastically modified the environments bees inhabit. The conversion of natural habitat into urban and agricultural spaces has created more fragmented and less species-rich habitats [44,101–103]. Reduced floral diversity and the loss of natural habitat are both key drivers of poor nutrition and reduced bee diversity [23,43–45,101]. Unsurprisingly, the forage diversity of flower-constant colonies was especially low in species-poor environments in our simulations. The *SDI* often approached zero as colony size increased and colonies used communication about high-quality flower types (figure 4). Flower constancy alone also had a negative impact on the *SDI*, but this effect was less strong in the absence of communication (approx. 5–20% lower *SDI* compared to indiscriminate colonies) (figures 3 and 4). Contrary to our expectation, habitat fragmentation did not reduce forage diversity further in flower-constant colonies (figure 5; but fragmentation reduced the number of food source visits by approx. 18–23%, electronic supplementary material). We compared two different types of food source clustering, a few large clusters versus many smaller clusters, and found that arranging food sources in many smaller clusters increased colony diet breadth by 10–30% compared to environments with fewer but larger clusters. In nature, bees often prefer to travel short distances between plants [104]. This preference is likely to further increase forage diversity when food sources are arranged in small clusters as bees will more often encounter heterospecific flowers in close proximity than when clusters are large. The effects of cluster size can be tested experimentally and should be considered when planning landscape modifications that aim to improve bee nutrition and health.

Flower constancy not only affects forage diversity but also impacts the energy collected by bees (which might be especially relevant for nectar foraging) because flower-constant bees risk bypassing more rewarding flower species [7,9,10]. Simulations suggest that flower constancy reduces the energy a bee collects in a range of ecological circumstances, especially when foraging conditions are poor [14]. Under these circumstances, low levels of flower constancy should be strongly selected for. However, if foraging conditions are favourable, flower-constant bees, especially social species with communication, can potentially collect more energy [14], leading to contrasting selection pressures based on energy versus nutritional needs. Whether, how and under which circumstances bees prioritize energy versus nutritional diversity requires further research in species with different social lifestyles.

## 5. Conclusion

Our data suggest that flower constancy is likely to extensively reduce forage diversity in many environments, especially when combined with communication. Flower-constant colonies will not only have a lower forage diversity but they might often collect less food overall as bees skip rewarding food sources that are not of the preferred species [7,9,14]. This adds further mystery to the question of why social bees are often highly flower constant (figure 6) [17]. A better understanding of the nutritional needs of bee species is needed to understand the choices and challenges that different bee species face in different environments [23]. Furthermore, research is needed to explore the sensory abilities of bees to assess pollen quality and identity, which would allow nurse bees and foragers to follow strategies that reduce the risks of nutritional imbalances [34,95–98].

## Data Availability

Simulation data can be found in the electronic supplementary material. The Netlogo code used for the different versions of the agent-based model to generate the data can be found here: [70]. Supplementary material is available online [105].
